# 

**Published:** 1862-04

**Authors:** 


### WORKS PUBLISHED DURING THE QUARTER.

### 1. *Medicine.*

BASHAM.—On Dropsy connected with Disease of the Kidneys (Morbus Brightii), and of some other Diseases of those Organs, associated with Albuminous and Purulent Urine. Illustrated by numerous Drawings from the Microscope. By W. R. Basham, M.D. Second Edition, 8vo, cloth, 9s.

CATLIN.—The Breath of Life; or, Mal-Respiration and its Effects upon the Enjoyments and Life of Man. By George Catlin. (Manu-graph.) With Illustrations. 8vo, 2s. 6d.

FRERICHS.—Atlas of Pathological Anatomy, illustrative of a Clinical Treatise on Diseases of the Liver. By F. T. Frerichs, M.D. Translated and edited by C. Murchison, M.D. 4to, bds. Part I. Second Edition revised, 15s. Part II. 17s. 6d.

GAIRDNER.—Public Health in Relation to Air and Water. By W. T. Gairdner, M.D. Fcap. 8vo. 7s. 6d.

HANKS.—On Teething of Infants; its Prevalent Errors, Neglects, and Dangers; their Influence on the Health, and as Causes of Death of Children, including the Dangers of Teething Powders, Soothing Powders, Soothing Syrups, &c. Illustrated by Cases. By Henry Hanks, L.R.C.P.E. (Exam.) M.R.C.S., &c. Fcap. 8vo, cloth, price 3s. 6d.

HASTINGS.—Inquiry into the Medicinal Value of the Excreta of Reptiles in Phthisis, and some other Diseases. By J. Hastings, M.D. Post 8vo, 5s.

HEALE.—A Treatise on the Physiological Anatomy of the Lungs. By James Newton Heale, M.D. With Engravings, 8vo, cloth, 8s.

LEE.—Homœopathy: A Rejoinder to the Replies to Sir B. Brodie's Letter to "Fraser's Magazine." Supplementary to the Fourth Edition of "Homœopathy and Hydropathy impartially appreciated." By Edwin Lee, M.D., &c. Post 8vo, 1s. 6d.

LITZMANN.—Contributions to the Knowledge of Osteomalacia. By C. C. T. Litzmann, M.D. Translated from the German by J. M. Duncan, M.D. 8vo, 1s. 6d.

MACKENZIE.—The Pathology and Treatment of Phlegmasia Dolens, as deduced from Clinical and Physiological Researches, being the Lettsomian Lectures on Midwifery, delivered before the Medical Society of London, during the Session 1861-2. By F. W. Mackenzie, M.D., M.R.C.P.L. 8vo, cloth, 6s.

The Medical Directories for 1862. Embracing in One Volume, the London and Provincial, Scotland, and Ireland. 8vo, cloth, 10s. 6d.

The Medical Register for 1862. Published under the direction of the General Council of Medical Education and Registration of the United Kingdom, pursuant to the Act to Regulate the Qualifications of Practitioners in Medicine and Surgery. Royal 8vo, cloth, 4s.

O'REILLY.—The Placenta, the Organic Nervous System, the Blood, the Oxygen, and the Animal Nervous System, physiologically examined. By John O'Reilly, M.D., F.R.C.S. Ireland. With Engravings, 8vo, cloth, 5s.

SANTA.—Climate of Algiers in Reference to the Chronic Affections of the Chest. By P. de Pietra Santa. 8vo, sewed, 1s. 6d.

SILVESTER.—The Physiological Method of inducing Respiration in Cases of Drowning, Still-Birth, Suffocation from Chloroform, &c. By Henry R. Silvester, M.D. Lond. Second Edition, post 8vo, 1s.

Transactions of the Epidemiological Society of London. Vol. I., part 2, 4s.

WALSHE.—Practical Treatise on the Diseases of the Heart and Great Vessels, including the Principles of Physical Diagnosis. By W. H. Walshe, M.D. Third Edition, revised and much enlarged. Post 8vo, 12s. 6d.

WEST.—Illustrations of Puerperal Diseases. By R. Uvedale West, M.D., F.R.C.S. Edin. 8vo, cloth, 3s. 6d.

WILSON.—On Diseases impeding Reproduction. By M. Wilson, M.D. 8vo, 5s.

WITT.—An Effectual and Simple Remedy for Scarlet Fever and Measles, with an Appendix of Cases. By Charles Witt, M.R.C.P. 8vo, sewed, 1s.

### 2. *Surgery.*

APPIA.—The Ambulance Surgeon, or Practical Observations on Gun-shot Wounds. By P. L. Appia, M.D. Edited, with Notes and short Descriptions of Surgical Appliances, by T. W. Nunn and A. M. Edwards. Fcap. 8vo, cloth, 6s.

BRYANT.—Clinical Surgery. Part III. The Surgery of the Mouth, Pharynx, Abdomen, and Rectum, including Hernia. By Thomas Bryant, F.R.C.S., Assistant-Surgeon to Guy's Hospital. 8vo, 3s. 6d.

GIBB.—On the Diseases and Injuries of the Hyoid or Tongue Bone. By George D. Gibb, M.D., M.R.C.P. 8vo, 2s. 6d.

HOLT.—On the Immediate Treatment of Stricture of the Urethra. By Barnard Holt, F.R.C.S. With a Plate, 8vo, cloth, 3s.

WILDE.—An Essay on the Malformations and Congenital Diseases of the Organs of Sight. By W. R. Wilde, M.D., F.R.C.S. With Sixty Engravings, 8vo, cloth, 7s. 6d.

### 3. *Chemistry.*

MILLER.—Elements of Chemistry, Theoretical and Practical. By W. A. Miller, M.D., F.R.S. Vol. III. Organic Chemistry. Second Edition, 8vo, cloth, 20s.

### THE CASE OF MR. W. F. WINDHAM.

We had proposed in the present number of the Journal, to have laid before our readers a critical analysis of the Windham case in its medico-legal aspects. We are induced, however, as the most legitimate method to be adopted for a correct appreciation of the issues involved, and the most just course to be pursued to all parties concerned in this important case, to postpone its consideration until the legal proceedings still pending in connexion with it have terminated.

